# Moderate-dose caffeine enhances anaerobic performance without altering hydration status

**DOI:** 10.3389/fnut.2024.1359999

**Published:** 2024-07-09

**Authors:** Ahmet Mor, Kürşat Acar, Dan Iulian Alexe, Hakkı Mor, Mekki Abdioğlu, Maria Cristina Man, Fatih Karakaș, Fatma Ben Waer, Ali Kerim Yılmaz, Cristina Ioana Alexe

**Affiliations:** ^1^Department of Coaching Education, Faculty of Sport Sciences, Sinop University, Sinop, Türkiye; ^2^Department of Physical Education and Sports, Faculty of Sport Sciences, Sinop University, Sinop, Türkiye; ^3^Department of Physical and Occupational Therapy, “Vasile Alecsandri” University of Bacau, Bacau, Romania; ^4^Department of Coaching Education, Yasar Doğu Faculty of Sport Sciences, Ondokuz Mayıs University, Samsun, Türkiye; ^5^Faculty of Sport Sciences, Institute of Health Sciences, Ankara University, Ankara, Türkiye; ^6^Department of Physical Education, 1 Decembrie 1918 University, Alba Iulia, Romania; ^7^Research Laboratory Education, Motricité, Sport et Santé (EM2S) LR19JS01, High Institute of Sport and Physical Education of Sfax, University of Sfax, Sfax, Tunisia; ^8^Recreation Department, Yasar Doğu Faculty of Sport Sciences, Ondokuz Mayıs University, Samsun, Türkiye; ^9^Department of Physical Education and Sports Performance, “Vasile Alecsandri” University of Bacau, Bacau, Romania

**Keywords:** sports nutrition, supplements, ergogenic aid, soccer, caffeine

## Abstract

The effects of direct nutritional supplements on athletic performance are still being investigated and arouse curiosity. Only one study in the literature was found that investigated the kicking speed performance of futsal players following low-dose caffeine supplementation (3 mg/kg); thus, the question of whether caffeine supplementation improves kicking speed as well as essential physical parameters in soccer players is still controversial. Therefore, the aim of this study was to determine the effect of caffeine supplementation on vertical jump (VJ), sprint, reaction time, balance, change of direction (COD), and ball-kicking speed in soccer players. In a double-blind, cross-over design, nine moderately trained male soccer players (21.11 ± 2.02 years, 171.22 ± 6.14 cm, 71.78 ± 10.02 kg) consumed caffeine (6 mg/kg) or a placebo 60 min before completing balance, reaction time, vertical jump, agility, 30 m sprint, and ball-kicking speed tests. Greater VJ height (*p* = 0.01) and power (*p* = 0.08), and faster completion time according to the Illinois Agility Test (*p* = 0.08) were found following caffeine supplementation compared to placebo. Elapsed time (*p* = 0.01), average (*p* = 0.01) time, and the slowest reaction times (*p* = 0.016) were significantly reduced after caffeine consumption compared to placebo supplementation. Caffeine intake significantly improved VJ, agility, and reaction time (*p* < 0.05) but did not affect 30 m sprint, ball-kicking speed, balance, and RPE values in soccer players (*p* > 0.05). Although non-significant, caffeine intake also improved sprint (0.67%) and ball kicking (2.7%) performance percentages. Also, caffeine consumption did not induce dehydration, and the athletes’ body hydration levels were normal. These findings support the use of caffeine supplementation as an effective nutritional ergogenic aid to enhance anaerobic performance, at least for vertical jumps, COD speed, and reaction time, in trained male soccer players.

## 1 Introduction

Soccer performance is characterized by bursts of high-intensity physical activity, which requires players to simultaneously perform intense running, and explosive soccer-specific actions such as kicking, jumping, sprinting, and agility (1). An ergogenic aid is defined as any intervention encompassing training techniques, mechanical devices, nutritional components, pharmacological methods, or psychological strategies that can enhance exercise performance capacity or improve training adaptations (2). Thus, the efficacy of ergogenic aids, such as caffeine, on anaerobic performance is of interest to soccer players, coaches, and sports scientists. Caffeine is the most widely used ergogenic aid by the athletic population (3). Indeed, following the removal of caffeine from the World Anti-Doping Agency’s list of banned substances, it has been reported that three of every four professional athletes use caffeine before or during competition (4). As such, in a study by Tallis et al. (5) 35 of the 36 clubs across the English professional soccer leagues reported that caffeine was administered to enhance soccer performance. The rationale behind this phenomenon is the underlying mechanisms by which the ergogenicity of caffeine may benefit anaerobic performance, which are the antagonism of the adenosine receptor at concentrations in the micromolar range in the central nervous system (CNS) (6), increased β-endorphins secretion via activation of the hypothalamic-pituitary-adrenal (HPA) axis (7), and intracellular calcium release from the sarcoplasmic reticulum in muscle cells (8), all of which can benefit performance via improving power, agility, reaction time, and alertness and delaying fatigue (6).

The ergogenic effect of caffeine supplementation has been reported in a variety of soccer-specific performance parameters, such as improved performance in the Loughborough Soccer Passing Test (9), the 20 m sprint test (10), fatigue resistance in 75% of the VO2max to volitional fatigue test (11), jumping performance in the countermovement jump (CMJ) test (11, 12), agility in the arrowhead agility test (10), and reaction time (10, 11, 13). However, direct extrapolation of these findings to the complexity of soccer is complicated, and no study has yet investigated all these parameters in the same test setting with the same participant groups (6). Since the ergogenicity of caffeine may be influenced by various factors, such as genotype (14), training status (15), habituation to caffeine (16), and supplementation regimen, various physical performance responses to caffeine can differ even in the same person (6). Therefore, evaluating the effect of caffeine supplementation on all these soccer-specific skills and/or parameters in the same field test battery is important for improving understanding of the potential of caffeine supplementation to soccer performance.

Kicking is another key skill and/or component of soccer. Indeed, ball-kicking speed has been suggested as a new, efficient performance indicator in youth soccer players (17). Given that the ball-kicking speed is associated with and/or affected by various physical aspects (e.g., technique, power, and balance), it might be anticipated that caffeine supplementation has the potential to influence ball-kicking speed; however, empirical evidence to support this is presently lacking. While one previous study by Lopez-Samanes et al. (18) reported no difference in ball velocity performance in futsal players after low-dose of caffeine (3 mg/kg) supplementation, it remains unknown whether caffeine supplementation improves the ball-kicking speed in soccer players. As such, further research is required to assess the effect of caffeine supplementation on the ball-kicking speed in soccer players. Therefore, the aim of this study was to determine the effect of moderate-dose (6 mg/kg) caffeine supplementation on vertical jump, sprint, reaction time, balance, agility, and ball-kicking speed in the same field test battery in moderately trained male soccer players. In addition, body hydration levels and fluid balance were analyzed to determine the effect of caffeine on body water homeostasis. It was hypothesized that (I) the ingestion of moderate-dose of caffeine would improve vertical jump, sprinting, reaction time, balance, agility, and ball-kicking speed and (II) not induce dehydration and fluid imbalances.

## 2 Materials and methods

### 2.1 Participants

Nine healthy, non-smoking, young, moderately trained (19), male soccer players participated in this study (age 21.11 ± 2.02 years, height 171.22 ± 6.14 cm, weight 71.78 ± 10.02, habitual consumption of caffeine 188 ± 83 mg d^−1^; mean ± SD, Table 1). The required sample size was estimated using G*Power software (Heinrich-Heine-University Düsseldorf, version 3.1.m9.2, Düsseldorf, Germany). A sample size of eight participants was determined sufficient (effect size: 0.50, confidence interval: 1-*β* 0.95, error: *α* 0.05, actual power: 0.96). Based on Acar et al. (20) and discussion between the authors, we set the effect size at 0.5. All participants had 9.89 ± 2.57 years of competitive soccer experience at club standard and at least 3 years of experience playing in regional and university-level leagues. Participants completed at least five weekly soccer training sessions (7.5 h a week). All participants declared that they had not used any ergogenic aids that might alter body hydration levels and exercise performance within 3 months from the start of the study. All participants were informed of the experimental procedures before giving their written informed consent.

**Table 1:** Descriptive information of subjects (*n* = 9).

Variables	X	SD
Age (yr)	21,11	2,02
Height (cm)	171,2	6,14
Weight (kg)	71,78	10
BMI (kg/m^2^)	24,37	1,9
Training age (yr)	9,89	2,57
Habitual consumption of caffeine (mg/day^−1^)	188	83

### 2.2 Experimental design

**Figure 1:**
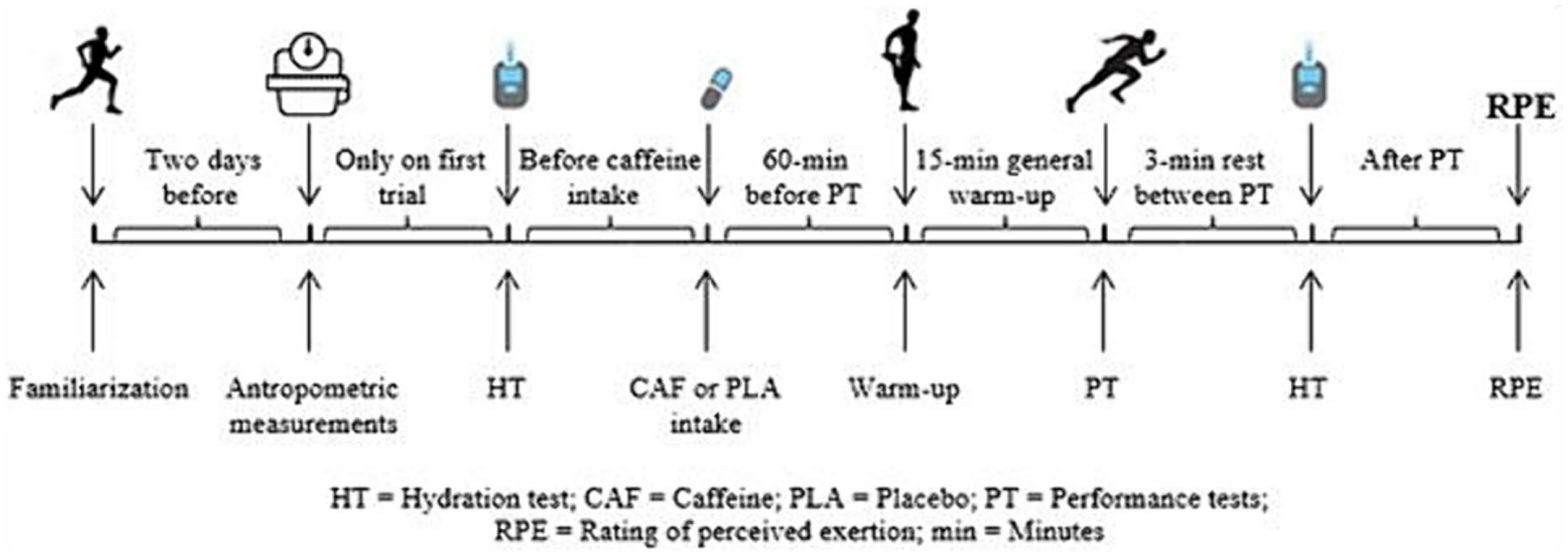
Schematic diagram of the experimental design.

On the first visit, a habitual caffeine consumption questionnaire was given to the participants in addition to their anthropometric and body composition assessments. Body mass was obtained in kg with a bioelectric impedance analysis device (BIA, Inbody 120, InBody Co., Ltd. Seoul, Korea), and height was obtained in cm with a portable stadiometer (Seca 213, Hamburg, Germany). Habitual caffeine intake was determined through a validated questionnaire (21). Only participants with a daily caffeine intake of less than 250 mg d^−1^ were included to control individual differences in responsiveness to caffeine from habituation. In addition, participants were selected based on the following inclusion criteria: (1) were aged between 18 and 25 years; (2) had actively participated in soccer training for at least 3 years, with a minimum of three times/week for the last 3 three months; (3) were non-smokers. On the first visit, participants also performed the testing protocol for familiarization at a low intensity that would not make them exert vigorous effort. Following completion of this initial familiarization, participants were assigned to ingest either caffeine or a placebo in a double-blind, cross-over, randomized counterbalanced design. The participants were randomly assigned to the two conditions using the Research Randomizer software (www.randomizer.org; accessed on 10 June 2022). Participants consumed 6 mg/kg of caffeine (Nature’s Supreme, Istanbul, Turkey) or placebo supplements (wheat bran) in capsules (same color and form, made up of gelatin hard form) 60 min before the testing protocol (5). A researcher, who had no further involvement in this research, prepared the caffeine dose using electronic laboratory scales with one milligram of sensitivity at room temperature. There were at least 48 h between sessions to ensure that the caffeine had washout and to allow participants to complete recovery. Participants were asked to record their diet 24 h before the first test session and replicate it 24 h before the second test session. For 24 h before and for each of the testing sessions, participants were asked to refrain from ingestion of caffeine, ergogenic aids (e.g., nitrate, sodium bicarbonate), alcohol, and anti-inflammatory drugs; not to engage in strenuous physical activity; and to be strict with their nutrition and rest. Regarding the rhythm (22), the tests and measurements were applied to the participants at the same time of the day (between 1–3 pm), under similar environmental conditions (ambient temperature 22.00 ± 1.41°C, humidity 62.00 ± 4.24%, pressure 1018.00 ± 1.41 mbar; mean ± SD) in the Sinop University indoor sports hall and performance laboratory. Participants were instructed to wear the same clothing and footwear for all the testing sessions. In the second and third sessions, participants’ hydration levels were analyzed just before placebo or caffeine ingestion and immediately after tests. Participants completed a 15 min standardized warm-up. *Ad libitum* water consumption (similar amounts) was allowed in both trials. The testing protocol in each experimental session consisted of balance, vertical jump, reaction time, change-of-direction via the Illinois Agility Test, 30 m sprint, and the ball-kicking speed tests, respectively. Vertical jump, reaction time, change of direction, 30 m sprint, and ball-kicking speed tests were performed in the indoor sports hall while balance, hydration levels, and RPE tests were conducted in the performance laboratory. These performance tests were employed because they were the same as soccer players’ moves in training and competitions. A 3 min passive rest period was given between the performance tests (except the vertical jump test) to facilitate recovery (23) and participants were allowed two trials with the best score used for subsequent analysis (24). In addition, the Borg CR10 scale with a range of 0–10 was used to measure the rate of perceived exertion (RPE) at the end of the tests (25). A schematic diagram of the experimental protocol is displayed in Figure 1.

### 2.3 Anthropometric and body composition assessments

Body mass was obtained in kg with a bioelectric impedance analysis device (BIA, Inbody 120, InBody Co., Ltd. Seoul, Korea), and stature was obtained in cm with a portable stadiometer (Seca 213, Hamburg, Germany).

### 2.4 Balance test

A portable dynamic balance device (Togu Challenge Disc 2.0, Prien am Chiemsee, Rosenheim, Germany) was utilized to assess the balance of the participants. The platform was free to move in all directions (up to a maximum of 12°) and thus provided an unstable ground. The challenge disc recorded the athlete’s movements with three-dimensional motion sensors and sent the data in real time to its software on the smartphone or tablet via Bluetooth. Stability index ranges were categorized into 1 to 5 (1—very good, 2—good, 3—normal, 4—weak, 5—very weak), and a lower score (*p*) indicated a better balance. Initially, the researcher showed the application on the tablet to the athlete at eye level, and the athlete stood barefoot on the platform to eliminate the possible effects of different types of shoes on the results. Later, the athletes were instructed to stand in the middle of the disc and keep their balance for 20 s (after 10 s of preparation, 5 s of which is a countdown) with their arms free to swing. During the test, participants were told to keep the point in the circle as central and stable as possible. The platform provided a safe measurement for athletes with its non-slip surface. The test was performed two times with a 3 minute passive rest, and the best score was used as the dynamic balance test score (26).

### 2.5 Reaction time test

Participants’ reaction times were determined using the Light Trainer (Reaction Development and Exercise System, Istanbul, Turkey). The reaction time test course consisted of four light modules (stuck to the top of 30 cm height traffic training cones) lined up side by side at a distance of 1 m. The test course was designed depending on the facility conditions (indoor sports hall) and the physical characteristics of the experimental group (trial with different numbers of modules and feedback). Athletes stood 1 m from the modules in the middle of the course. Participants got ready for the reaction time test on the “ready” command. The athletes deactivated the light modules, which were lit on the right or left side, with precise movements, touching with their dominant hand to the top of the module. The test started with activating the first light automatically and ended with the athletes’ “deactivating” the last light. Athletes were asked to deactivate 30 light modules in total. The test was repeated if the participants hit the modules, dropped them, or deformed the test course. The test was performed two times with a 3 minute passive rest, and the best score was used as the reaction time test score (27).

### 2.6 Vertical jump and anaerobic power test

A digital vertical jump device (Takei 5,406 Jump-MD Vertical Jumpmeter, Tokyo, Japan) was used to measure the vertical jump scores of the participants. Firstly, the rubber vertical jump plate was placed on a flat surface. In order to eliminate the possible effects of different types of shoes on the results, the participants were instructed to take off their shoes and stand “ready” with bare feet centered on the plate (10–20 cm from each other). Afterward, the researcher (the same person fastened the digital belt in all trials for test reliability) zeroed the digital belt, wound it tightly around to the waist of the participants, and turned the pulley gently in the direction of the arrow to take the slack out of the rope. Once the athletes were ready, they quickly moved from the upright standing position to a position of 90° flexion of the knees with freely swinging arms and jumped for the maximum height. The test was repeated if participants jumped by stepping forward, the measuring tape was loose, or they did not land on the rubber plate after jumping. Each player performed two trials interspersed with 1 min rest between each vertical jump, and the best (highest) jump was recorded in cm with an accuracy of ±1 (28). Participants’ anaerobic power calculations were executed using the Lewis formula: anaerobic power (W) = {√4.9 [body weight (kg)] √vertical jump (m)} (29).

### 2.7 Illinois agility test

The COD was assessed with the Illinois Agility Test by using a photocell (Seven, SE-165 Photocell Stopwatch, Istanbul, Turkey). The agility course is an area of 10 m long and 5 m wide, formed by four 30 cm height traffic training cones lined up in a straight line at 3.3 m intervals from each other in the middle. The test consists of a 40 m straight, and a 20 m slalom run with 180° turns every 10 m. The photocell timing gates were placed at the start and finish lines at a height of approximately 1 m. When they were ready, athletes started the test 30 cm behind the starting point. The participants were asked to run at maximal speed. The test was repeated two times with a 3 minute passive rest between each trial, and the best value was recorded as the Illinois Agility Test time (30).

### 2.8 30 M sprint test

The linear sprint times of the soccer players were determined with the 30 meter sprint test by using a photocell (±0.01 s precision) device (Seven, SE-165 Photocell Stopwatch, Istanbul, Turkey) in the indoor sports hall. When participants were ready, they started the sprint from a line one meter behind the starting gate with a standing start position. Participants were asked to run at maximum speed and performed the test twice with a 3 minute rest between trials. Test results were recorded in seconds and milliseconds, and the best values were used as 30 m sprint test time (27).

### 2.9 Ball-kicking speed test

The ball-kicking speed was determined 11 meters (penalty mark) from the goal using a radar gun device (Bushnell Velocity Speed Gun, Overland Park, Kansas, United States), which can measure speed in the range of 16–177 km/h with a sensitivity of ±2 km/h. Initially, the dominant legs of the participants were recorded (subjectively determined); thus, maximal speed was ensured. Then, participants shot the ball with the instep kick technique. Kicks were performed with a ball (size 5 soccer ball for 12 years and older) in compliance with FIFA standards. The researcher (the same person aimed the radar gun in all trials for test reliability) measured the ball-kicking speed behind the goal, directly opposite the penalty spot where the soccer player kicked the ball. Participants were told to kick for accuracy (hitting the target) while attaining the maximum ball-kicking speed. Each player was given two trials to get the best score, and the results were recorded in km/h (31).

### 2.10 Supplementation protocol

Participants consumed 6 mg/kg of caffeine (Nature’s Supreme, Istanbul, Turkey) or placebo supplements (wheat bran) in capsules (same color and form, made up of gelatin hard form) 60 min before the testing protocol (6). A researcher, who had no further involvement in this research, prepared the caffeine dose using electronic laboratory scales with one milligram of sensitivity at room temperature.

### 2.11 Hydration analysis

The portable hydration test device (MX3 Diagnostics LAB Pro, Melbourne, Australia) was used to assess participants’ body hydration levels (32). The device was convenient and easy to use for outdoor measurements and analyzed the hydration level from the saliva taken directly from the tongue with a hydration test strip. The saliva sample was taken from the tongue under the sterilization rules. The saliva sample was collected and analyzed with a hydration test strip attached to the device without waiting or undergoing any other procedure. The values and their assigned hydration status follow as ≤65 = Hydrated, 65–100 = Mildly Dehydrated, 101–150 = Moderately Dehydrated, >150 = Severely Dehydrated.

### 2.12 Statistical analysis

Data were checked for normality by using the Shapiro–Wilk test. Comparison between groups was analyzed with the paired sample *t*-test to test for differences between the caffeine and placebo supplement in the vertical jump, sprint, reaction time, balance, agility, and ball-kicking speed performances. Cohen’s *d* was utilized in the calculation of effect size (large *d* > 0.8, moderate *d* = 0.8 to 0.5, small *d* = 0.5 to 0.2, and trivial *d* < 0.2) (33). Statistical significance was accepted as *p* < 0.05, and all data were analyzed using SPSS 27.0 (IBM Corp., Armonk, NY) and are presented as mean ± SD.

## 3 Results

**Figure 2:**
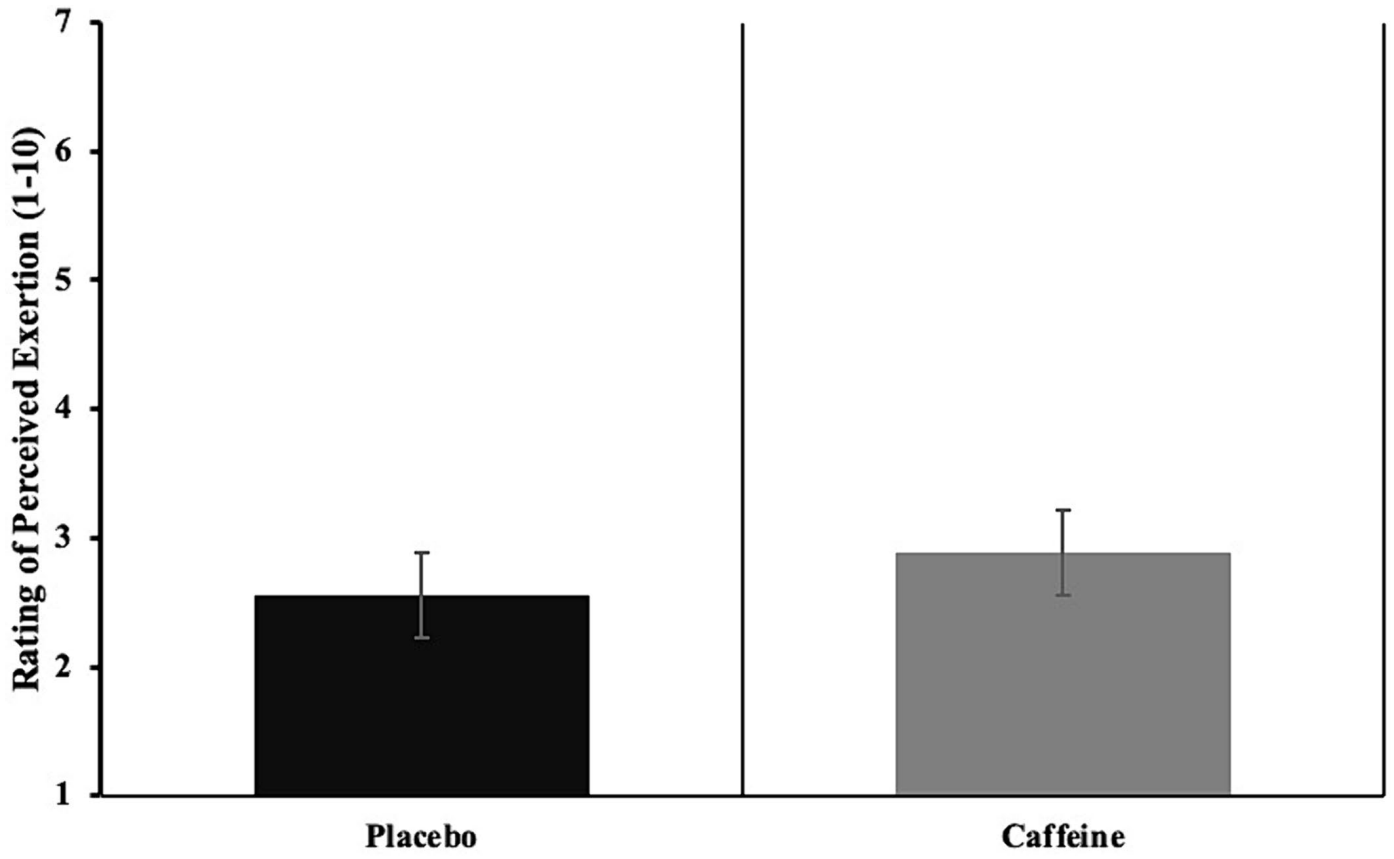
Rating of perceived exertion (RPE) (A.U).

Vertical jump height (*p* = 0.001, *d* = 0.83, Table 2) and power (*p* = 0.008, *d* = 0.45, Table 2) were significantly increased after caffeine compared to placebo supplementation. The Illinois Agility Test completion time was significantly faster after caffeine compared to placebo supplementation (*p* = 0.008, *d* = 1.20, Table 2). There was no difference in the 30 m sprint (caffeine: 4.39 ± 0.14 vs. placebo: 4.42 ± 0.20 s, *p* = 0.426, *d* = 0.17), balance (caffeine: 2.75 ± 0.48 vs. placebo: 2.60 ± 0.76 s, *p* = 0.624, *d* = 0.23) and ball-kicking speed values (caffeine: 93.87 ± 6.87 vs. placebo: 91.37 ± 5.13 km/h, *p* = 0.193, *d* = 0.41) between supplementations (Table 2). RPE values were also similar between caffeine (2.89 ± 0.33 AU) and placebo (2.56 ± 0.72 AU) supplementations (*p* = 0.195, *d* = 0.58) (Figure 2).

**Table 2:** Changes in mean values of caffeine and placebo groups.

Variables	Placebo	Caffeine	95% CI		*d*	*p*
	Х ± SD	Х ± SD	LB	UB		
VJ (cm)	56.33 ± 6.87	61.44 ± 5.24	−7.30	−2.92	0.83	0.001*
VJ (watt)	1183.45 ± 105.15	1241.29 ± 146.74	−95.8	−19.87	0.45	0.008*
Balance (s)	2.60 ± 0.76	2.75 ± 0.48	−0.05	0.10	0.23	0.624
30 m sprint (s)	4.42 ± 0.20	4.39 ± 0.14	−0.87	0.55	0.17	0.426
COD (s)	16.43 ± 0.42	15.97 ± 0.34	0.16	0.76	1.20	0.008*
Ball-kicking speed (km/h)	91.37 ± 5.13	93.87 ± 6.87	−6.57	1.56	0.41	0.193

*(*p* < 0,05); X, mean; SD, standard deviation; *d*, Cohen’s *d* effect size; VJ, vertical jump; COD, change of direction (Illionis agility test); 95% CI, confidence interval; LB, lower bound; UB, upper bound.

Regarding reaction time performance parameters, there was a significantly faster elapsed time (*p* = 0.001, *d* = 1.27, Table 3), average reaction time (*p* = 0.001, *d* = 1.37, Table 3), and the slowest reaction time (*p* = 0.016, *d* = 1.52, Table 3) after caffeine compared to placebo supplementation. However, there was no significant difference in the fastest reaction time (caffeine: 0.71 ± 0.08 vs. placebo: 0.74 ± 0.08 s, *p* = 0.238, *d* = 0.37) and in the last reaction time (caffeine: 0.95 ± 0.27 vs. placebo: 1.13 ± 0.30 s, *p* = 0.097, *d* = 0.63) between supplementations.

**Table 3:** Participants’ reaction time performance parameters.

Variables	Placebo	Caffeine	95% CI		*d*	*p*
	X ± SD	Х ± SD	LB	UB		
Elapsed time (s)	34.79 ± 1.71	31.92 ± 2.69	1.50	4.24	1.27	0.001*
Average reaction time (s)	1.16 ± 0.05	1.06 ± 0.09	0.06	0.14	1.37	0.001*
Fastest reaction time (s)	0.74 ± 0.08	0.71 ± 0.08	−0.04	0.41	0.37	0.238
Slowest reaction time (s)	1.96 ± 0.31	1.58 ± 0.17	−0.03	0.10	1.52	0.016*
Last reaction time (s)	1.13 ± 0.30	0.95 ± 0.27	0.10	0.65	0.63	0.097

*(*p* < 0.05); X, mean; SD, standard deviation; *d*, Cohen’s *d* effect size; 95% CI, confidence interval; LB, lower bound; UB, upper bound.

Fluid balance was within normal ranges in both groups, and there was no major effect on hydration levels between pre-and post-exercise conditions (placebo: 53.22 ± 16.57 mOsm/L vs. placebo: 54.78 ± 16.64 mOsm/L, *p* = 0.863, *d* = 0.09) (caffeine: 54.44 ± 20.92 mOsm/L vs. caffeine: 57.33 ± 20.79 mOsm/L, *p* = 0.715, *d* = 0.13) (Table 4).

**Table 4:** Hydration levels in both groups between pre- and post-exercise conditions.

Variables	Pre-exercise	Post-exercise	95% CI		*d*	*p*
	Х ± SD (mOsm/l)	Х ± SD (mOsm/l)	LB	UB		
Placebo	53.22 ± 16.57	54.78 ± 16.64	−1.96	−1.15	0.09	0.863
Caffeine	54.44 ± 20.92	57.33 ± 20.79	−3.60	−2.18	0.13	0.715

*(*p* < 0.05); X, mean; SD, standard deviation; *d*, Cohen’s *d* effect size; 95% CI, confidence interval; LB, lower bound; UB, upper bound.

## 4 Discussion

The aim of this study was to determine the effect of moderate-dose caffeine supplementation on vertical jump, sprint, reaction time, balance, agility, and ball-kicking speed in the same field test battery in moderately trained male soccer players. To the best of our knowledge, the current study is the first to analyze the effect of acute caffeine intake on ball-kicking speed, balance, sprint, agility, vertical jump, reaction time, and hydration status in the same test setting in male soccer players. The main finding was that the ingestion of 6 mg/kg of caffeine significantly enhanced vertical jump, agility, and reaction performance. However, no significant differences between placebo and caffeine were detected in hydration status, ball-kicking speed, balance, sprint performance, and rating of perceived exertion values. These findings are partly in line with our experimental hypothesis and support a moderate dose of caffeine supplementation as an ergogenic aid to enhance anaerobic performance, including vertical jump height, COD speed, and reaction time for male soccer players.

The current study ascertained that 6 mg/kg of caffeine increased vertical jump and change of direction performance, which is parallel to previous studies (9, 34, 35) and is in contrast to the other in which the participants were recreationally active young males who were not habituated to caffeine (36). This lack of significant difference in the abovementioned study may have resulted from the participants’ status as non-responders to caffeine. However, a performance improvement was seen with the caffeine for 47% of the participants during the 20-yard shuttle. Our findings significantly extend the observations of those previous studies and support the ergogenic potential of caffeine supplementation on vertical jump and change of direction performance. The positive effect on jumping performance might be related to increased motor unit recruitment (37) and muscle activation (38). Accordingly, caffeine improves performance through two primary mechanisms: antagonism of adenosine receptors (A1, A2A) in the central nervous system, which leads to increases in neurotransmitter release and potentiation of Na^++^/K^++^ pump activity in skeletal muscle, which may lead to an increase in excitation-contraction coupling (6). This mechanism of caffeine may also have enhanced jumping performance. In the study of futsal players, López-Samanes et al. (18) examined the effects of acute caffeine on physical performance. In light of the data obtained, the researchers suggested that acute caffeine supplementation significantly improved vertical jump performance. Nevertheless, although the researchers also found an increase in ball velocity, they did not find any significance. Ranchordas et al. (39) examined the effects of caffeinated gum on vertical jump, sprint, and recovery levels in soccer players, and they noted that 200 mg of caffeine slightly improved physical performance tests such as jumping and recovery. In line with these results, acute caffeine ingestion of 5 mg/kg 60 min before exercise was found to increase jump height in professional soccer players (12). Notably, the researchers found that a caffeine dose of 3 mg/kg improved performance in the majority of the post-exercise tests (10). These results, along with our findings, support the ergogenic effect of acute caffeine supplementation on jumping performance as it was well established in previous studies (40–42). This effect of caffeine on jumping performance could be attributed to the improvements in force production after caffeine ingestion (6). Evidently, caffeine ingestion has been well-documented to enhance peak power and mean power and reduce the time needed to reach peak power in the Wingate test (43). Also, it has been reported that caffeine decreases contraction time and maximal displacement values, which indicates an increase in muscle contraction performance. More importantly, this study was carried out using tensiomyography, in which an electrical pulse induces muscle contraction independent of the CNS. So, it could be considered compelling evidence of caffeine’s direct effect on neuromuscular stimulation (44). The improvement in COD time in the present study might be attributed to the “blocking adenosine” mechanism, which increases neurotransmission (45) and motor unit recruitment (46). This change can be elucidated by the fact that caffeine intake increases Ca + and thus improves mobility by facilitating muscle contraction and nerve conduction (47). Similarly, in their study with rugby players, Ranchordas et al. (48) investigated the effects of caffeinated gum on CMJ, Illinois Agility, 6 × 30 m repeated sprint, and Yo-Yo IR2 test performances. Research results indicated that caffeinated gum augmented performance in the Yo-Yo IR2 and the CMJ tests and decreased the fatigue index during repeated sprints. Furthermore, Karayigit et al. (49) reported that low (3 mg/kg) and moderate (6 mg/kg) doses of caffeinated coffee improved repeated sprint performance with increasing epinephrine norepinephrine concentrations in female team sport athletes. In contrast, it was determined that 6 mg/kg acute caffeine consumption did not affect agility and anaerobic power (50). Lastly, it has already been known that chronic exposure to caffeine may result in physiological modifications that lead to tolerance and reduce the ergogenic effects of acute caffeine on high-intensity exercise (16). Because the habitual caffeine intake level of the participants in the current study was not high (188 ± 83 mg/day), the tolerance phenomenon did not appear in this research.

Considering the present study’s balance and reaction time performance findings, it was statistically determined that caffeine intake decreased the reaction time, thus increasing the reaction performance. Caffeine has an adenosine-like molecular structure and binds to the adenosine receptor (AA2) in the brain, increasing the concentration of neurotransmitters (51). Also, caffeine is a central nervous system stimulant due to its capacity to block adenosine-specific receptors, which increases the release of several neurotransmitters, including norepinephrine, dopamine, acetylcholine, and serotonin (52). Accordingly, the significant difference in reaction time may have concluded from motivators through hormonal changes. In two similar studies, Impey et al. (53, 54) reported that caffeine consumed 60 min prior to a performance in soccer goalkeepers positively affected reaction time. Similarly, it was shown that both 3 and 6 mg/kg of acute caffeine intake enhanced reaction time in female team sport athletes (55). These decreases in reaction time could be associated with the mechanism of caffeine as an adenosine receptor antagonist (6), which may enhance neuronal excitability that leads to improvements in sport-specific reaction times (56). Moreover, in our study, increase in reaction performance may be responsible for the decrease in change of direction time via the same mechanism that also makes motor unit recruitment easier. Hence, caffeine may play a significant role in individual and team sports where concentration and reaction times influence match/training performance. To the contrary, Bottoms et al. (56) showed that 3 mg/kg caffeine intake had no effect on reaction time in athletes while Balko et al. (57) suggested that a larger amount of caffeine may lead to a decrease in visual and auditory reaction times, in turn, increasing reaction time performance. Interestingly, some results are inconsistent with our findings, most likely because the present study enrolled different levels of soccer players than the studies above. The participants in our study were amateur soccer players with various training modalities and experiences. Therefore, these inconsistencies in findings may be primarily associated with the fact that they are not familiar with the high demand standardized exercises like professional soccer players and thus have a different level of responsiveness to tests. Furthermore, according to previous studies, acute caffeine consumption may improve (58) or diminish (59) the standing balance ability of young adults. This discrepancy may be partially attributable to the variance in caffeine dosage provided, which ranges from 160 to 400 mg. Only Kara et al. (60) gave a caffeine dosage according to body mass (6 mg/kg); this was the only research that found positive results. Given the paucity of published research evaluating the influence of caffeine on balance, it is difficult to contextualize these results in light of prior research. In addition, methodological inconsistencies and changes in the balancing tasks and outcome measures used may possibly account for the inconsistent results. There is a need for a more thorough and extensive evaluation of the effects of caffeine on balance skills, based on the small amount of data and inconsistent results.

The findings of our study indicated that 6 mg/kg caffeine consumption did not induce dehydration, and the athletes’ body hydration levels were normal. Reviews have shown that this is a common result, suggesting that no caffeine-induced dehydration or other harmful changes occur in athletes during exercise that negatively affects physical performance (61, 62). Furthermore, Del Coso et al. (63) reported that acute caffeine consumption of 6 mg/kg increased urine flow and sweat electrolyte excretion; however, these effects were not enough to affect dehydration or blood electrolyte levels when exercising for 120 min in a warm environment. These results, consistent with our study’s findings, refute the widespread belief that caffeine consumption is accompanied by dehydration and an increased sweat rate in the body. Physiologically, the effects of arginine vasopressin on water retention and the effects of aldosterone on sodium balance seem to be sufficient to tackle the effects of a mild diuretic consumed in a moderate dose (61). Considering RPE values, caffeine is supposed to elicit ergogenic effects on the CNS via the antagonism of adenosine receptors, leading to pain suppression, which may attenuate the pain and decrease the RPE (6). Additionally, along with its particular chemical structure, caffeine easily crosses the blood–brain barrier, acts on adenosine receptors (A1, A2), and blocks the receptors that cause pain in the body. This is another factor expected to be effective on RPE values in the present study (64). Intriguingly, we did not observe a significant impact of caffeine on RPE levels, indicating that other variables, such as greater motor neuron activation and a reduced decline in voluntary activation throughout the exercise, may explain caffeine’s ergogenic benefits.

The following are some of the limitations of our study. The blinding efficiency was not evaluated by asking individuals to identify which supplement (caffeine vs. placebo) they had ingested. Unknown is whether “caffeine expectancy” may have influenced the outcomes of the present investigation. Although participants were advised to repeat their 24 h meal before each test, macronutrient consumption was not evaluated. In addition, we did not collect samples for determining neurotransmitter concentrations at the baseline and post-exercise, and we did not assess the electromyographic activation of active muscles during performance tests, which would have offered greater insight into the specific processes by which caffeine boosted reaction time, vertical jump, and change of direction performance but not sprint, balance, and ball-kicking performance.

## 5 Conclusion

The present study found that a 6 mg/kg caffeine intake in soccer players improved physical performance, such as vertical jump height, change of direction speed, and reaction time. Although non-significant, caffeine intake also improved sprint (0.67%) and ball kicking (2.7%) performance percentages. Moreover, it was revealed that caffeine did not impair body hydration levels, so body fluid balance was within normal clinical ranges. Accordingly, further studies conducted with larger sample sizes using a 24 h urine collection method could provide more evidence for the relationship between caffeine consumption and hydration. On the other hand, in our study, caffeine ingestion did not provide any change in balance performance and rating of perceived exertion. Based on these results, it is recommended that coaches and athletes incorporate caffeine into their nutritional strategies as it can improve performance. In future studies, the effects of different doses of caffeine on various components of performance, including small-sided games and ball-kicking speed in soccer and other disciplines, could be investigated to provide new insights. Ultimately, to verify these mechanisms, further studies should be conducted to investigate neuromuscular responses to caffeine supplementation during anaerobic tasks in soccer players.

## Data availability statement

The raw data supporting the conclusions of this article will be made available by the authors, without undue reservation.

## Ethics statement

The studies involving humans were approved by Human Research Ethics Committee at Sinop University (Reference number: E-57452775-050.01.04-104421). The studies were conducted in accordance with the local legislation and institutional requirements. The participants provided their written informed consent to participate in this study.

## Author contributions

AM: Conceptualization, Data curation, Formal analysis, Methodology, Project administration, Supervision, Writing – original draft, Writing – review & editing. KA: Data curation, Formal analysis, Writing – review & editing. DA: Funding acquisition, Methodology, Project administration, Software, Supervision, Writing – review & editing. HM: Methodology, Writing – original draft, Writing – review & editing. MA: Project administration, Supervision, Writing – original draft, Writing – review & editing. MM: Data curation, Funding acquisition, Software, Visualization, Writing – review & editing. FK: Data curation, Writing – review & editing. FW: Funding acquisition, Software, Visualization, Writing – review & editing. AY: Formal analysis, Supervision, Writing – review & editing. CA: Data curation, Funding acquisition, Software, Supervision, Visualization, Writing – review & editing.
